# 

**Published:** 1892-02-20

**Authors:** 


					February 20th, 189
WITH EXTRA NURSING SUPPLEMENT.
Per Annum, 6s. 6d., post free.
Monthly Farts, 6d.; post free, 8d.
Ditto per Annum, 8s., post free
~?TTHO MA S S H OS PI TAL
WD Si OR IT/CH HOSPITAL
.ascoit V/esTiew Infirmary"
WITH EXTRA NURSING SUPPLEMENT.
No. 282. Vol. XI.] Satttrday, February 20th, 1892. [One Peknt.

				

